# Management of a Parturient with Mast Cell Activation Syndrome: An Anesthesiologist's Experience

**DOI:** 10.1155/2018/8920921

**Published:** 2018-05-22

**Authors:** Sangeeta Kumaraswami, Gabriel Farkas

**Affiliations:** Department of Anesthesiology, New York Medical College at Westchester Medical Center, Valhalla, NY, USA

## Abstract

Mast cell activation syndrome (MCAS) is a disorder in which patients experience symptoms and signs attributable to inappropriate mast cell activation and mediator release. Multiorgan involvement in patients can result in significant morbidity and possible mortality. Limited literature exists regarding anesthetic management of patients with MCAS. We report a case of vaginal delivery with neuraxial labor analgesia in a parturient with this condition and highlight the importance of multidisciplinary planning for uneventful outcomes. Stress can trigger life-threatening symptoms, and counseling is important to allay patients' fears. Optimum medical control, adequate premedication, avoidance of triggers, and preparedness to treat serious mediator effects are key. We review MCAS and discuss anesthetic considerations for patients with this mast cell disorder.

## 1. Introduction

Mast cell activation syndrome (MCAS) is a condition in which patients experience recurrent and episodic symptoms of mast cell degranulation. Patients with this disorder appear to represent a growing proportion of the mast cell disorder patient population [1, 2]. There is paucity of literature on this subject relevant to an anesthesiologist. Our pregnant patient had symptomatology suggestive of a mast cell mediated disorder with significant suffering and disability. Written consent was taken from her for publication of this case report.

## 2. Case

A 24-year-old female G3P2 was admitted for induction of labor at 39 weeks' gestation. Her pregnancy had been uneventful except for a diagnosis of gestational hypertension (diet controlled). During a scheduled obstetric visit at 39 weeks, she met criteria for preeclampsia with resulting hospitalization. She had history of 2 prior vaginal deliveries with neuraxial analgesia, both following induction of labor for preeclampsia.

Her medical history was significant for iron deficiency anemia (received iron infusions for treatment), hypothyroidism (controlled with levothyroxine), celiac disease, and a recent diagnosis of MCAS. A few months after the birth of her second child 2 years ago, she began experiencing potentially life-threatening reactions. These reactions typically followed a pattern of abdominal discomfort and diarrhea followed by extremity and facial swelling, hives, and throat itching. Triggers included certain foods, drugs, and physical or emotional stress. The patient reported frequent emergency room (ER) visits for this condition. Self-administration of epinephrine autoinjector and diphenhydramine resulted in relief of symptoms during most episodes. Occasional treatment with corticosteroids was necessary without need for overt resuscitative measures related to fluid replacement or airway management. A treatment regimen for MCAS was started and avoidance of known triggers including nonsteroidal anti-inflammatory drugs (NSAIDs) and histamine-rich foods was advised. Her surgical history included a bone marrow biopsy, esophagogastroduodenoscopy, and colonoscopy with no complications. Her physical examination was significant for obesity (body mass index 33) and an unremarkable back and airway examination.

During routine obstetric visits, she repeatedly requested elective cesarean delivery with general anesthesia due to concern for allergic reactions during labor. Our obstetric team saw no contraindication to a vaginal delivery and after intense counseling including discussion with an anesthesiologist she agreed to a trial of labor with early neuraxial analgesia. A multidisciplinary approach involving an obstetrician, anesthesiologist, hematologist, allergist, neonatologist, nursing personnel, and dietician was used to formulate a plan for delivery. A premedication regimen prior to delivery, with availability of an anaphylaxis treatment kit and resuscitation equipment at bedside, was planned. In addition to the existing daily regimen of cetirizine, famotidine, and montelukast, she was informed to start prednisone 60 mg daily about 48 hours prior to estimated date of delivery. Low-histamine and gluten-free diet precautions were to be followed during her hospital stay.

Following induction of labor with a Foley transcervical balloon and vaginal misoprostol, she requested epidural analgesia. Doses of 125 mg methylprednisolone and 50 mg diphenhydramine were given intravenously as premedication, and the procedure was accomplished with good pain relief. After approximately 4 hours, she complained of increasing pain secondary to uterine contractions. Examination of her lumbar area revealed a dislodged epidural catheter due to unclear reasons. The epidural procedure was repeated after again administering a prophylactic dose of 50 mg diphenhydramine, and catheter placement was done uneventfully. Alcohol based chlorhexidine was used uneventfully for skin antisepsis both times. She received 0.2% ropivacaine infusion through the epidural catheter with adequate pain relief. Oxytocin was used for augmentation of labor with delivery of a healthy neonate without complications. Her postpartum hospital stay was complicated by an unusual episode of abdominal discomfort and itchy throat, after exposure to odor from a citrus fruit (histamine-rich food), consumed by the other patient in her shared room. She was immediately given 50 mg diphenhydramine with resolution of symptoms and shifted to a single occupancy room. Our patient was discharged home on the third day following delivery without any other complications.

## 3. Discussion

Mast cells are an important part of our body's immune system, originating from the bone marrow and participating in inflammatory processes with production of mediators [3]. A certain level of mast cell activation is physiological and necessary for maintenance of homeostasis [4]. Mast cell activation syndromes is an umbrella term used to describe disorders in which recurrent and inappropriate mast cell activation and release of mediators occurs, causing symptoms associated with multiple organ systems.

The term was introduced to propose a global unifying classification of all mast cell activation disorders, with division into primary (proliferation of abnormal mast cells), secondary (normal mast cells activated in response to a microenvironmental trigger), and idiopathic (no evidence of primary or secondary cause) as shown in Table 1 [1, 5].

### 3.1. Presentation and Diagnosis

Typical symptoms and signs are listed in Table 2 [6].

Proposed diagnostic criteria for MCAS are listed in Table 3 [1].

Mast cells can be activated through both IgE and non-IgE dependent mechanisms with release of mediators such as histamine, tryptase, leukotrienes, and prostaglandins. Activation typically occurs in response to triggers, although none may be identified [7]. Clinical manifestations occur secondary to tissue responses to these mediators.

Our patient had episodic symptoms throughout her pregnancy with no disorder identified that could account for them. A bone marrow biopsy done had shown no evidence of mast cell disease, despite some abnormalities in megakaryocyte clustering and reticulum fibrosis. An elevated serum tryptase was found during an ER visit, with normal serum tryptase and blood histamine level in between episodes. With known primary and secondary causes ruled out, a potential diagnosis of idiopathic MCAS was made based on diagnostic criteria. A thorough workup was deferred until after delivery. Patients are often known to undergo extensive medical evaluation to determine an etiology, with a goal to find a yet-to-be identified endogenous or environmental stimulus or mast cell defect.

### 3.2. Treatment

Avoidance of exposure to identifiable triggers and antimediator therapy, including medications that prevent mast cell degranulation, form the basis of treatment. Multidrug therapy, such as H-1 and H-2 receptor antagonists, mast cell stabilizers, and leukotriene receptor antagonists are used in varying combinations to achieve control. Epinephrine autoinjector and antihistamine drugs are typically used by patients for breakthrough degranulation, with more aggressive treatment done in a hospital setting if necessary. Recently, omalizumab has been shown to prevent symptoms and reduce adverse events. Cytoreductive and immunomodulating therapies are tried in some cases.

### 3.3. Anesthetic Considerations

Little is known about the perioperative management of patients with MCAS. The main anesthetic concern is avoidance of mast cell mediator release. In addition to workup for evaluation of comorbidities, a multidisciplinary plan for perioperative management with involvement of the patient is necessary to lessen concerns. Medications taken for MCAS should be continued up to the day of surgery. A premedication regimen of H-1 and H-2 receptor antagonists and corticosteroids are recommended before invasive procedures including those requiring anesthesia, the goal being reduction and blockade of mediators that can cause life-threatening reactions such as anaphylaxis [8]. Benzodiazepines are valuable in reducing anxiety, a known trigger.

Deviation from routine anesthetic techniques is not necessarily warranted although central and peripheral neuraxial techniques reduce risk of multiple drug administration [9]. Adequate premedication, avoidance of triggers, and emergency preparedness are key. A list of possible perioperative triggers is shown in Table 4 [10]. Judicious use and increased vigilance are mandatory if such triggers cannot be avoided.

Data on adverse drug reactions and mast cell disease is scarce. Knowledge of drugs that can cause histamine release is key, with avoidance suggested based on theoretical assumptions. Drugs which are suspected to have caused previous reactions should be avoided [9].

Preprocedural skin testing is not recommended unless a previously documented hypersensitivity reaction exists [11]. Patients may experience reactions to medications they have tolerated previously. With limited knowledge of causative mechanisms, vigilance is key. Usage of perioperative drugs in this context is described in Table 5 [8, 10–14].

Serious perioperative reactions caused by mast cell mediators can be anaphylactic or anaphylactoid. Occurrence of such reactions is likely to be higher as compared to the general population. Clinical features mainly involve the skin and cardiovascular and respiratory systems. Management should focus on withdrawing the offending agent, interrupting the effects of the mediators already released, and preventing more mediator release. Symptomatic and supportive treatment include oxygen, H-1 and H-2 antagonists, corticosteroids, bronchodilators, epinephrine, fluids, and airway resuscitation [10]. Measurement of serum mediators (e.g., tryptase) during the episode, with identification and testing of all exposures, should be done to determine etiology, although results are often negative or insufficiently reliable.

### 3.4. MCAS and Pregnancy

Data from studies on pregnancy and delivery in patients with mast cell disorders is reassuring [10, 15]. Mast cells exhibit a beneficial function in pregnancy by contributing to implantation, placentation, and fetal growth. Excessive release of mediators can be associated with preterm delivery. Although the use of systemic treatment should be limited or even avoided in pregnancy, optimum management is recommended for maternal and fetal well-being. With appropriate medical control, there is no contraindication to pregnancy [16]. Vaginal delivery with early neuraxial analgesia is permissible, in the absence of an obstetric indication for cesarean section. Similar perioperative considerations apply for either mode of delivery. Practitioners should be aware of possible sedation in the newborn when H-1 antagonists are used directly before delivery.

## 4. Conclusion

MCAS is an area of ongoing research. Our patient had an uneventful pregnancy, labor, and delivery despite the increased morbidity from MCAS. At 3 months' postpartum, she continued to report frequent allergic reactions and currently follows up with a mast cell disorders specialist. With this review, we attempt to add to the limited anesthesia literature regarding MCAS. Knowledge of this condition with appropriate planning and preparation will help ensure optimal outcomes.

## Figures and Tables

**Table 1:** Classification of disorders associated with mast cell activation^*∗*^.

PRIMARY	SECONDARY	IDIOPATHIC
(i) Clonal mast cell disorders (e.g., mastocytosis) (ii) Monoclonal mast cell activation syndrome	(i) Allergic disorders (e.g., asthma, rhinitis) (ii) Mast cell activation associated with inflammatory or neoplastic disorders (iii) Physical urticaria (iv) Chronic autoimmune urticaria	(i) Urticaria (ii) Angioedema (iii) Anaphylaxis (iv) Mast cell activation syndrome(MCAS)^†^

^*∗*†^The term MCAS has been used interchangeably in the literature to denote both the umbrella term and idiopathic MCAS [4, 17].

**Table 2:** Common symptoms and signs of MCAS.

Dermatologic	Flushing, pruritus, hives
Cardiovascular	Near syncope or syncope, palpitations, chest pain, dysrhythmias, hypotension, hypertension
Pulmonary	Cough, wheezing
Eyes, ear, nose, throat	Post nasal drip, inflammation (conjunctivitis, rhinitis, sinusitis, pharyngitis, laryngitis), throat itching and swelling
Neurologic	Headache, seizures, tremors
Psychiatric	Cognitive dysfunction, memory difficulties, anxiety, depression, psychoses
Gastrointestinal	Nausea, vomiting, reflux, constipation, diarrhea, abdominal pain, malabsorption
Musculoskeletal	Bone or muscle pain, arthritis, myositis
Immunologic	Types I, II, III, and IV hypersensitivity reaction

**Table 3:** Proposed diagnostic criteria for MCAS.

(1) Episodic symptoms of mast cell mediator release involving ≥2 organ systems
(2) Appropriate response to antimediator therapy
(3) Documented increase in validated systemic markers of mast cell activation during episode (e.g., serum tryptase or urinary markers such as histamine metabolites, prostaglandin D2 or its metabolite, and leukotriene E4)
(4) Primary and secondary causes ruled out

**Table 4:** Perioperative triggers and treatments.

Type	Stressor	Treatment
Psychological	Anxiety, emotional stress	Pharmacologic, quiet environment

Mechanical	Pressure (tourniquet and BP cuff), friction (tape) surgery (GI tract a rich source of mast cells)	Minimize operative time, optimal positioning,
	Pain	Multimodal analgesia

Pharmacologic	Histamine releasing drugs	Avoid

Temperature	Hypothermia, hyperthermia, change in temperature	Heat maintenance devices, warm environment, warm intravenous and irrigation fluids

Infection	Bacterial, viral, fungal	As necessary

Foods, odors	Histamine-rich foods, odors (food, perfumes)	Avoid, single occupancy room

BP: blood pressure and GI: gastrointestinal.

**Table 5:** Perioperative drugs and mast cell disease^a^.

Class	Drug	Usage in mast cell disorders
Hypnotic/sedative agents	Propofol^b^, dexmedetomidine, etomidate, ketamine^b^	Acceptable
	Methohexital, thiopental	Thiopental causes histamine release

Inhalational anesthetics	Halogenated (isoflurane, sevoflurane, desflurane), nitrous oxide	Acceptable

Benzodiazepines^b^	Midazolam, diazepam	Acceptable

Opioids^c^	Morphine, meperidine, codeine	Causes histamine release
	Hydromorphone, fentanyl, sufentanil, alfentanil, remifentanil, buprenorphine	Acceptable

Nonopioid analgesics	Acetaminophen	Acceptable
	NSAIDs (ketorolac, nefopam)	Causes overproduction of leukotrienes (a mast cell mediator)

Neuromuscular blocking agents	Depolarizing NMBA (succinylcholine)	Acceptable
	Nondepolarizing aminosteroids^c^ (rocuronium, vecuronium^b^ pancuronium)	Acceptable
	Nondepolarizing benzylisoquinolines (atracurium, mivacurium, cisatracurium)	Atracurium and mivacurium cause histamine release

Reversal of neuromuscular blockade	Neostigmine, sugammadex	Acceptable

Local anesthetics^c^	Amides and esters	Acceptable

Antiseptics^c^	Alcohol, chlorhexidine, povidone-iodine	Acceptable

Intravenous fluids	Crystalloids, colloids, albumin, gelatin, hydroxyethyl starch^c^	Acceptable

Common labor and delivery drugs	Oxytocin, prostaglandins, methylergonovine, tocolytic agent (terbutaline)	Acceptable, though role of prostaglandins in causing or worsening reactions is unclear

Antibiotics^c^	Penicillins, cephalosporins, sulfonamides, vancomycin, polymyxin B, clindamycin, fluoroquinolones	Vancomycin and polymyxin B can cause histamine release

Miscellaneous	Adenosine, atropine, glycopyrrolate, ondansetron, beta-blockers, ACEI, protamine, aprotinin (fibrin glue), blood transfusion, dyes, contrast media, and latex^c^	Acceptable; adenosine and protamine can cause histamine release; beta-blockers can attenuate the effect of epinephrine in anaphylaxis; ACEI can augment an anaphylactic reaction

NSAIDs: nonsteroidal anti-inflammatory drugs; NMBA: neuromuscular blocking agents; ACEI: angiotensin converting enzyme inhibitors. ^a^Drugs associated with histamine release should be avoided if another equally effective drug can be used; alternatively, they must be administered slowly. ^b^Drugs reported to cause in vitro histamine release from human mast cells. ^c^Drugs and products associated with high incidence of hypersensitivity reactions in the general population do not need to be avoided unless a previously documented sensitivity exists.
